# Robust DNA Damage Response and Elevated Reactive Oxygen Species in *TINF2*-Mutated Dyskeratosis Congenita Cells

**DOI:** 10.1371/journal.pone.0148793

**Published:** 2016-02-09

**Authors:** Larisa Pereboeva, Meredith Hubbard, Frederick D. Goldman, Erik R. Westin

**Affiliations:** 1 Department of Medicine, Division of Hematology Oncology, University of Alabama at Birmingham, Birmingham, Alabama, United States of America; 2 Department of Pediatrics, Division of Hematology Oncology, University of Alabama, Birmingham, Alabama, United States of America; Tulane University Health Sciences Center, UNITED STATES

## Abstract

Dyskeratosis Congenita (DC) is an inherited multisystem premature aging disorder with characteristic skin and mucosal findings as well as a predisposition to cancer and bone marrow failure. DC arises due to gene mutations associated with the telomerase complex or telomere maintenance, resulting in critically shortened telomeres. The pathogenesis of DC, as well as several congenital bone marrow failure (BMF) syndromes, converges on the DNA damage response (DDR) pathway and subsequent elevation of reactive oxygen species (ROS). Historically, DC patients have had poor outcomes following bone marrow transplantation (BMT), perhaps as a consequence of an underlying DNA hypersensitivity to cytotoxic agents. Previously, we demonstrated an activated DDR and increased ROS, augmented by chemotherapy and radiation, in somatic cells isolated from DC patients with a mutation in the RNA component of telomerase, *TERC*. The current study was undertaken to determine whether previous findings related to ROS and DDR in *TERC* patients’ cells could be extended to other DC mutations. Of particular interest was whether an antioxidant approach could counter increased ROS and decrease DC pathologies. To test this, we examined lymphocytes from DC patients from different DC mutations (*TERT*, *TINF2*, *and TERC*) for the presence of an active DDR and increased ROS. All DC mutations led to increased steady-state p53 (2-fold to 10-fold) and ROS (1.5-fold to 2-fold). Upon exposure to ionizing radiation (XRT), DC cells increased in both DDR and ROS to a significant degree. Exposing DC cells to hydrogen peroxide also revealed that DC cells maintain a significant oxidant burden compared to controls (1.5-fold to 3-fold). DC cell culture supplemented with N-acetylcysteine, or alternatively grown in low oxygen, afforded significant proliferative benefits (proliferation: maximum 2-fold increase; NAC: 5-fold p53 decrease; low oxygen: maximum 3.5-fold p53 decrease). Together, our data supports a mechanism whereby telomerase deficiency and subsequent shortened telomeres initiate a DDR and create a pro-oxidant environment, especially in cells carrying the *TINF2* mutations. Finally, the ameliorative effects of antioxidants *in vitro* suggest this could translate to therapeutic benefits in DC patients.

## Introduction

The clinical manifestations of Dyskeratosis Congenita (DC) are due to insufficient telomere maintenance within cells, resulting in critically shortened telomeres. In its classical form, DC is characterized by a mucocutaneous triad of abnormal skin pigmentation, nail dystrophy, and leukoplakia, as well as a predisposition to bone marrow failure, pulmonary fibrosis, and cancer[1]. To date, DC mutations have been found in eleven telomere/telomerase related genes (*DKC1*, *TERT*, *TERC*, *TINF2*, *TCAB1*, *CTC1*, *NOP10*, *USB1*, *RTEL1*, *NHP2*) and recently *PARN*[2–4]. Telomeres are composed of hexameric DNA repeats (TTAGGG) found at chromosomal termini that are maintained and elongated by the ribonucleotide enzymatic complex telomerase. Telomerase is minimally composed of a catalytic component, TERT and an RNA template, TERC. Telomerase is thought to be recruited to the telomere by TPP1 and TINF2, which are part of a protein complex called shelterin that facilitates the formation of a telomeric secondary structure (T-loop). This secondary structure alters chromosome termini from stimulating a double-strand DNA damage response (DDR) and subsequent cellular responses. Past evidence suggests decreased telomerase activity and prematurely shortened telomeres exhaust stem cell pools, exemplified by progressive marrow failure in DC[5, 6]. Understanding mechanisms by which shortened telomeres lead to DC pathogenesis will be crucial in predicting disease outcome and designing therapeutic interventions.

Under steady-state conditions, telomeres form a secondary structure that evades DNA damage surveillance, while shortened and dysfunctional telomeres trigger a double-stranded DNA repair mechanisms[7]. Briefly, this process begins with the local deposition of 53BP1/γH2AX followed by a signaling cascade that includes ATM/ATR, CHK1/2 resulting in the activation of the tumor suppressor p53. This sustained p53 activity related to telomere dysfunction in turn leads to replicative senescence or apoptosis in affected cells. Manifestations of several BMF syndromes also have an underlying DDR component related to their pathogenesis. Causative mutations in Fanconi’s anemia (FA), Diamond-Blackfan anemia (DBA), myelodysplastic syndrome (MDS) and 5q- syndrome all have been demonstrated to mobilize p53 that disrupts hematopoiesis[8–10]. The role of p53 activation in DC has also been confirmed in murine models of DC (Dkc1 Δ15[11], *TRF2*[12]) and primary human cells (*DKC1*, *TERT*, *TERC*)[13, 14]. Highly replicative tissues (bone marrow, intestinal epithelium, and skin) and stem cells require telomere maintenance for long-term survival and thus are sites of clinical manifestations in DC[15]. Intolerance to p53 activation may play a role in disease pathogenesis, as heightened p53 activation in DC cells is also a recognized contributor to the pro-apoptotic state and inherent sensitivity of these cells to DNA damaging agents associated with bone marrow transplant (BMT) regimens[16].

We previously demonstrated an association between elevated DDR and ROS that led to diminished cell function and proliferation in various cell types isolated from *TERC*-mutated DC patients that could be rescued with antioxidant approaches[14, 17, 18]. Abrogation of the DDR pathway (TERT overexpression or p53/p21-shRNA) similarly decreased ROS and/or increased cell proliferation; conversely, ROS could be elicited via telomere disruption in normal cells[17]. Herein, we have undertaken studies to further investigate whether elevated ROS and DDR are consistent among other causative DC mutations. Findings of elevated ROS in patients’ cells harboring *TERT*, *TINF2* and *TERC* mutations were consistent and likely coincident with the degree of DDR activation suggesting these assays may serve as adjunctive tests in disease diagnosis. Finally, our ability to downregulate DDR with NAC and decreased oxygen exposure provides a potential mechanistic and therapeutic insight towards treating the systemic manifestations of this condition.

## Material and Methods

### Patients

**Fig 1:**
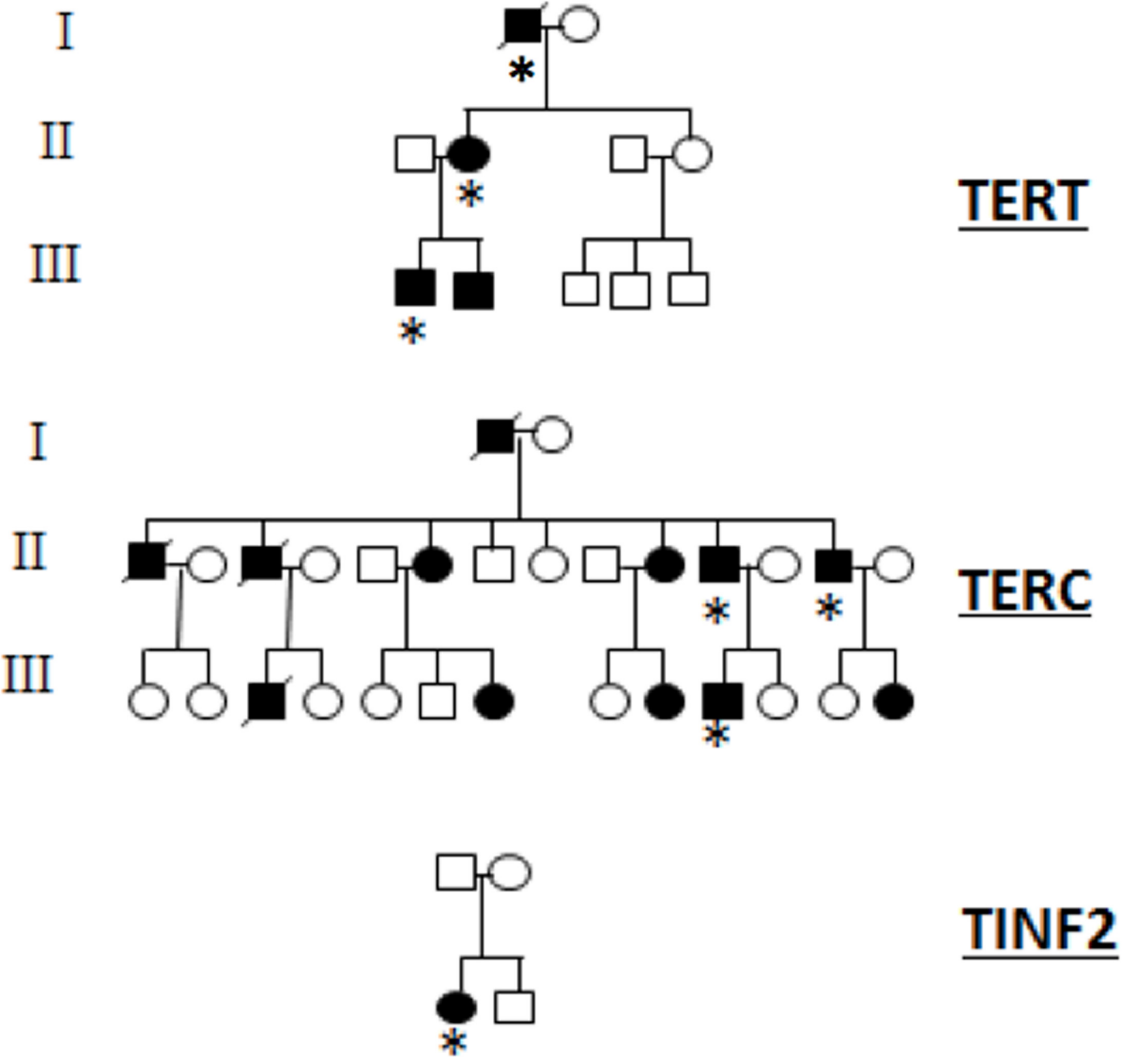
TERT, TERC and TINF2 pedigrees. Pedigrees for *TERT*, *TERC* and *TINF2* DC patients included within this study. Affected individuals are highlighted (filled circles/squares) and individuals who provided cells for this study (asterisk).

Blood samples and clinical information were obtained from DC patients and healthy volunteers. All participants provided their written informed consent to participate in this study, which was approved by the University of Alabama at Birmingham Internal Review Board (F100512004). Cells used in our study were obtained from DC patients with following underlying heterozygous mutations: *TERC* (451bp deletion incorporating the terminal 74 base pairs of the *TERC* gene; 3 patients)[19]; *TERT*, R631W mutation (3 patients); *TINF2*, R282C mutation (1 patient) (Fig 1).

### Cells and tissue culture

Mononuclear cell fractions were isolated from blood following Histopaque-1077 (Sigma Aldrich) gradient separation and frozen in aliquots. When possible, experiments were performed on freshly isolated patients’ cells. Thawed cells were cultured in complete RPMI-1640 media (10% fetal calf serum, 1000 U/ml penicillin and streptomycin, 20mM L-glutamine) supplemented with 50 U/mL human interleukin-2 (IL2, Peprotech). T-cells were activated via CD3/CD28 Dynabeads (Invitrogen) added at a 1:1 bead-to-cell ratio on Day 1 to promote cell growth. Cell counts were performed on the Cellometer Auto T-4 automated cell count and viability analyzer (Nexcelom Bioscience).

### Induction/rescue of DNA damage and oxidative stress

DNA damage was induced by single exposure (100–500 cGy) using X-ray irradiation (XRT; ionizing radiation; X-RAD 320, Precision X-Ray Inc. North Branford, CT). To increase oxidative stress, cell cultures were supplemented with hydrogen peroxide at varying concentrations (1-100uM) for 30 minutes. To alleviate ROS, cells were treated with 5mM N-acetylcysteine (NAC; Sigma Aldrich) for varying time periods or by culturing cells in low oxygen (1%) (Biospherix hypoxia chamber).

### Measurement of intracellular ROS

The presence of ROS was measured by dichlorofluorescin diacetate (DCF-DA, Sigma) staining followed by fluorescence-activated cell sorting (FACS) or alternatively plate-based detection of fluorescence. For FACS-based DCF detection, cells were collected at indicated times, washed with PBS and incubated in 10uM DCF-DA for 10 minutes at 37°C. After washing twice with PBS, cells were subjected to FACS analysis. ROS levels were quantified by recording the mean fluorescent intensity (MFI). Flow cytometry was performed using a BD FACSCalibur and results were analyzed using CellQuest software. Larger oxidative stress experiments utilized a plate reader as described in Roesslein *et al*. with slight modifications[20]. Briefly, lymphocytes were collected and treated with 1, 10 or 100uM H_2_O_2_ at 37°C for 30 minutes. After PBS washing, the cells were counted and stained with DCF-DA as described above at a concentration of 5x10^5^ cell/ml followed by distribution in triplicate to wells of a black 96-well plate at 1x10^5^ cells/well. To control for variation in cell numbers, each cell sample was stained in parallel with calcein (1 mg/ml) to quantify the proportion of viable cells. Fluorescent intensities were read using a fluorescence microplate reader (BioTeck Instruments) at 485/528 nm excitation/emission wavelengths.

### Western blotting

Standard western blotting techniques were used as previously described[17, 21]. Briefly, cells were pelleted and lysed with Complete Lysis-M buffer (Roche). Whole cell extracts were subjected to SDS-PAGE electrophoresis, transferred to a nitrocellulose membrane, and stained with the following antibodies: p53 (Calbiochem), p53S15 (serine-15), p-ATM (serine-1981), p-BRCA1 (serine-1524), γH2AX (serine-139) (phosphorylated antibodies from Cell Signaling) and Actin (GenScript), followed by the corresponding secondary antibody conjugated with HRP (Santa Cruz).

### Statistical analyses

Student’s t-test was applied to assess statistical significance between two groups of data and reported by calculated p-values. Analysis was performed using Graphpad Prizm software. Error bars within graphs are representative of the standard deviation of DC or control samples in each experiment.

## Results

### Increased basal and radiation-induced p53 in DC lymphocytes

**Fig 2:**
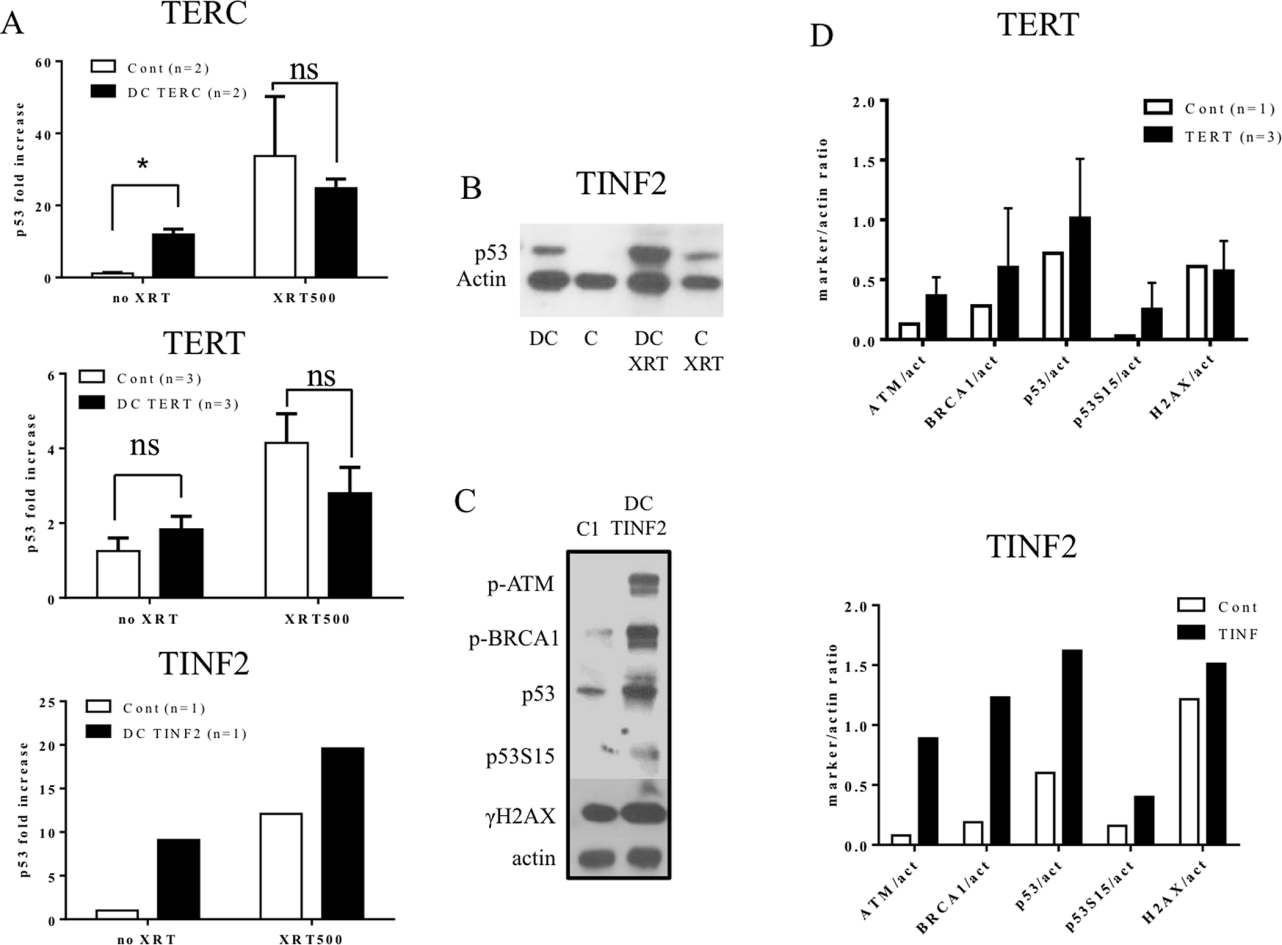
Basal and radiation-induced DDR Response in DC lymphocytes. Control and DC lymphocytes were cultured for 5–6 days, irradiated with 500 cGy and collected after 24 hours (XRT: ionizing radiation). (A) p53 western blots were quantified by densitometry for TERC, TERT and TINF2 patients. Average increases in p53 were found in all untreated DC cells while only TERC cells revealed a statistical increase (p<0.01). Radiation increased p53 in all cells with no significant increase in DC cells vs controls. (B) A representative TINF2 blot demonstrating heightened p53 response pre- and post-irradiation. (C) A representative TINF2 blot presenting heightened steady-state DDR. (D) Phosphorylated ATM, BRCA1, p53 and γH2AX were quantified by densitometry. Data within each graph (2A, D) is calculated by the densitometry of the respective DDR value divided by the actin densitometry value. Error bars represent standard deviation of each patient subset (TERC: 2 patients, TERT: 3 patients, TINF2: 1 patient, and their respective controls). Statistics not provided for TINF2 cells due to availability of only a single patient. Data is representative of three experimental replicates.

We have previously reported increased p53 under routine culture conditions in *TERC*-mutated lymphocytes. Here, we extended our investigation to examine whether DC cells harboring non-*TERC* DC mutations (*TERT* and *TINF2*) also display similar properties. When averaging DC *TERC* (two patients), *TERT* (three patients) and *TINF2* (one patient) p53 increases, in general, demonstrated an average 2–10 fold increase of steady-state p53 compared to controls (Fig 2A and 2B; *TERC*: p<0.01; *TERT*: not significant; *TINF2*: no statistics due to single patient). As expected, p53 increased in all DC samples and controls following XRT (Fig 2A and 2B; XRT: ionizing radiation = 500cGy). *TERT* and *TERC* cells consistently acquired higher p53 levels than controls under steady-state and XRT-conditions yet the magnitude of the p53 increase was greater in control cells. This is in contrast to *TINF2* cells that revealed a more robust response than controls providing evidence that variability exists among the DC cells in relation to DDR. While the degree of telomere shortening was similar in all the DC mutations tested (data not shown), a wide range of clinical pathologies was observed. Of note, the *TINF2* patient, despite her young age, had advanced clinical symptomatology (Table 1). We previously characterized p53 responses and uncovered significant increases in total and phosphorylated p53 in *TERC* lymphocytes[14]. We performed similar western blots on p53 and additional markers consistent with DDR (p-ATM, p-BRCA1, p-p53, γH2AX) and found upregulation in *TINF2* and *TERT* cells relative to controls (Fig 2C and 2D). Inter-patient variation was found among the three TERT patients as represented by larger error bars (Fig 2C) with two patients indicating an increase in DDR and one patient decreased (*TERT* changes in p53 were 2-fold, 1.5-fold and 0.7-fold). This trend was consistent among three experiments. *TINF2* and *TERT* cells both reveal an increase in nearly all DDR measures yet *TINF2* cells are distinguished by a more robust steady-state DDR (Fig 2C and 2D). These results are consistent with our previous studies and indicate that DC lymphocytes with different mutations have a heightened steady-state DDR but may differ in the degree of their respective DDR and response to XRT.

**Table 1:** Mutations and clinical phenotypes of DC patients in the study.

Pt Code	Inheritance	Age/ generation	Sex	Clinical Phenotype	Hematological Findings	Telomere Length	ROS/DDR Trend Summary
*TERC-1*		42/II	M	G,D,S,O	-	short	ROS ↑ DDR ↑
*TERC-2*	AD	12/III	M	D	T	short	ROS ↑ DDR ↑
*TERC-3*		35/II	M	G,D,S	A,N,T	short	ROS ↑^B^ DDR ↑^B^
*TERT-1*		35/II	F	G,S,D	-	short	ROS ↑^C^ DDR ↔
*TERT-2*	AD	11/III	M	D	T	short	ROS ↑^C^ DDR ↔
*TERT-3*		60/I	M	G,D,S,O,L	A,N,T	short	ROS ↑ DDR ↑
*TINF2*	AD^A^	12/?	F	D,S,O,R	T	short	ROS ↑↑ DDR ↑↑

Substantial variations in clinical and hematological finding were observed between individual patients, however, short telomeres and increased ROS/DDR were a common feature of all DC patients irrespective of a mutation. Mutations include: TERC (451bp deletion; includes 74bp 3’ terminus), TERT (c.1891C > T R631W) and TINF2 (c.844C > T R282C). Clinical and hematological findings include: premature grey hair (G), dyskeratotic nails (D), skin dyspigmentation (S), leukemia (L), oral changes (O), pulmonary disease (P), anemia (A), neutropenia (N), thrombocytopenia (T) and retinopathy (R).

^A^indicates likely autosomal dominant.

^B^data reported in Pereboeva *et al*, PLoS One 2013.

^C^indicates ROS is elevated but variable.

### Basal and radiation-induced ROS increased in DC lymphocytes

**Fig 3:**
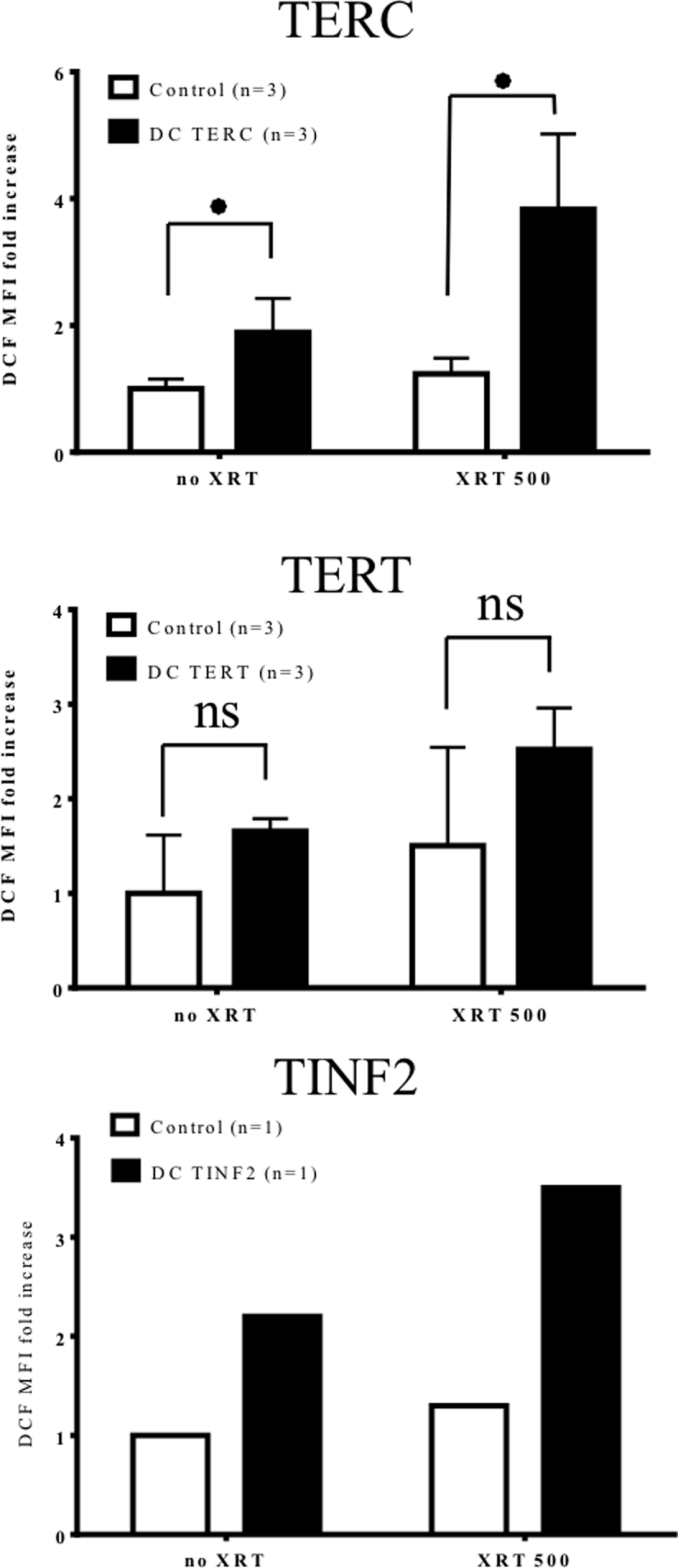
Basal and radiation-induced ROS in DC lymphocytes. Control and DC lymphocytes were cultured for 5–6 days and irradiated with 500 cGy. Basal (no XRT) and irradiated (XRT500) levels of ROS were determined by flow cytometry 24 hours post-irradiation via DCF staining. ROS levels are presented as the mean fluorescent intensity (DCF MFI) fold increase compared to the average value of non-irradiated control. Average increases in ROS were found in all untreated and treated DC cells compared to controls while only TERC cells reached statistical significance (untreated p = 0.050, irradiated p = 0.044). The error bars represent inter-patient variations in DCF staining of cultured lymphocytes in patients and controls. The cumulative data of three experiments is presented. Statistics not provided for TINF2 cells due to availability of only a single patient.

Elevated ROS was first reported by us in DC *TERC* fibroblasts, keratinocytes and lymphocytes[14, 17] and more recently by others[17, 22], as well as in cells from a DC mouse models[23, 24]. Given the consistent increase in DDR within lymphocytes from each of the DC mutations tested, we wanted to determine whether this trend extended to ROS. DC lymphocytes carrying *TERC*, *TERT* or *TINF2* mutations were grown in parallel with control lymphocytes and treated with DCF to assess steady-state ROS levels. DC *TERC* and *TINF2* cells had an approximate two-fold increase in steady-state ROS relative to controls while *TERT* cells had 1.5-fold increase (Fig 3; *TERC*: p = 0.05; *TERT*: not significant; *TINF2*: not statistics due to single patient). These cells were then exposed to 500cGy irradiation leading to further ROS production in DC cells (approximately two-fold; *TERC*: p = 0.044, *TERT*: not significant; *TINF2*: no statistics due to single patient) with negligible increases within controls suggesting distinct redox-related differences in response to XRT. Together these results demonstrate that DC lymphocytes harbor an average increase in steady-state ROS compared to controls that was further exacerbated post-XRT. Consistent with DDR results, ROS levels appeared to be stratified by severity with ROS levels highest in *TINF2* cells, followed by *TERC* and finally *TERT* (Fig 2).

### DC lymphocytes’ response to increased ROS

**Fig 4:**
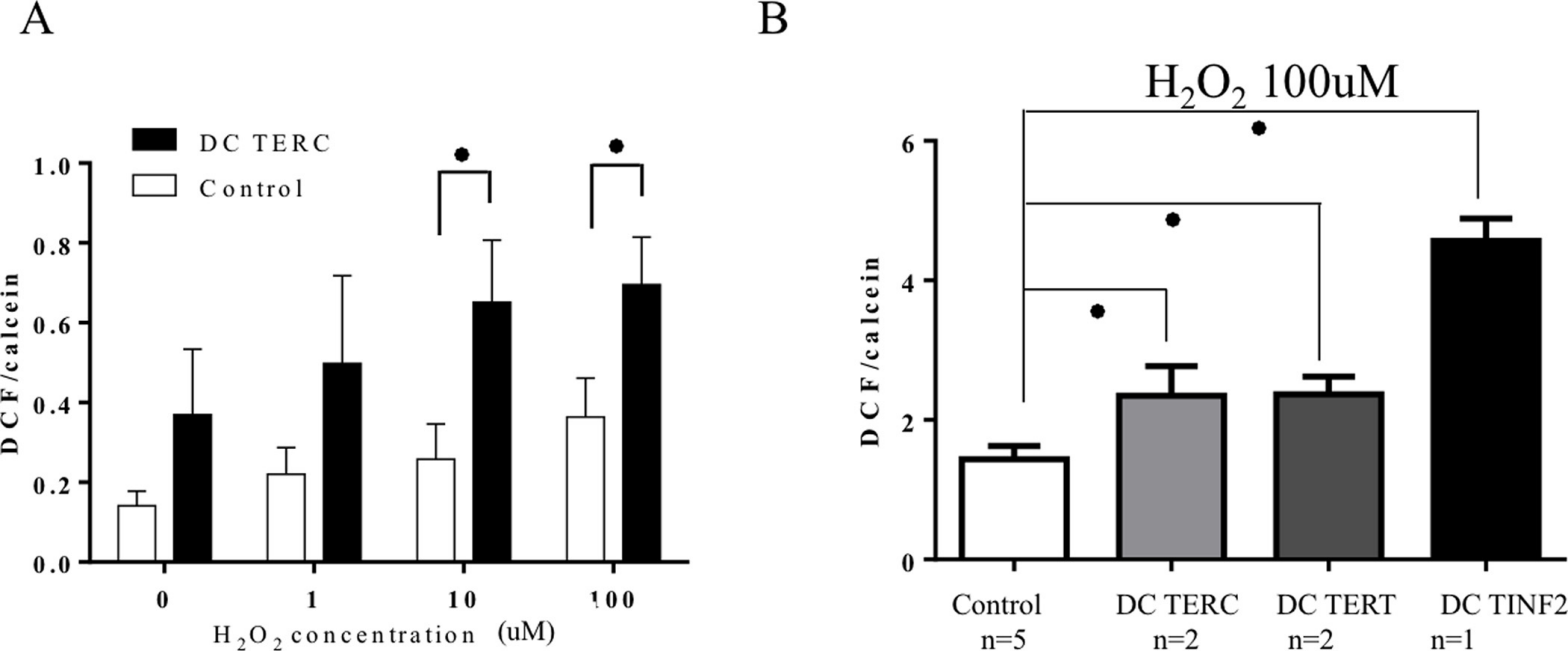
The effect of exogenous ROS on DC lymphocytes. DC and control lymphocytes were cultured for five days, treated with DCF and plated in triplicates in 96 well plates (50,000 cells/well). Samples were subjected to 30 minutes of the indicated peroxide concentrations (0-100uM), DCF fluorescence was measured by fluorescence plate reader and expressed as ratio of DCF/calcein staining. (A) Representative dose-dependent increase in ROS within control and TERC patient. Statistically significant differences in ROS levels were found at 10uM (p = 0.02) and 100uM peroxide (p = 0.036). Error bars presented experimental replicates. (B) ROS levels in DC and control lymphocytes in response to 100uM peroxide treatment. All DC cells demonstrated higher ROS compared to controls. Error bars present experimental replicates for each patient group. Unpaired t-test performed on control vs TERC, TERT and TINF2 found p<0.0001 in all cases.

Experiments outlined above evaluated ROS under steady-state and irradiation-induced conditions. We extended this investigation into ROS by evaluating these cells response to an exogenous source of ROS. To this end, *TERC* cells were cultured with increasing concentrations of hydrogen peroxide (1-100uM; Fig 4A). Supplementation of peroxide to cell culture resulted in a statistically significant, dose-dependent increase in ROS for both *TERC* and control lymphocytes, though higher levels were sustained in DC cells at all doses. At the highest peroxide concentration (100uM), all DC lymphocytes displayed significantly higher ROS levels compared to controls (Fig 4B). *TERC/TERT* cells had a two-fold increase compared to untreated cells however *TINF2* cells acquired a marked increase in ROS, distinct from DC counterparts (greater than three-fold; p<0.0001 in all cases). Again, *TINF2* cells appeared to have a more robust response to an exogenous stress compared to other DC cells. Together, these results suggest *TERT* and *TERC* cells may detoxify peroxide with kinetics similar to those of controls yet *TINF2* cells may be more susceptible to this particular exogenous source.

### Proliferation, ROS production and DDR expression in response to antioxidants

**Fig 5:**
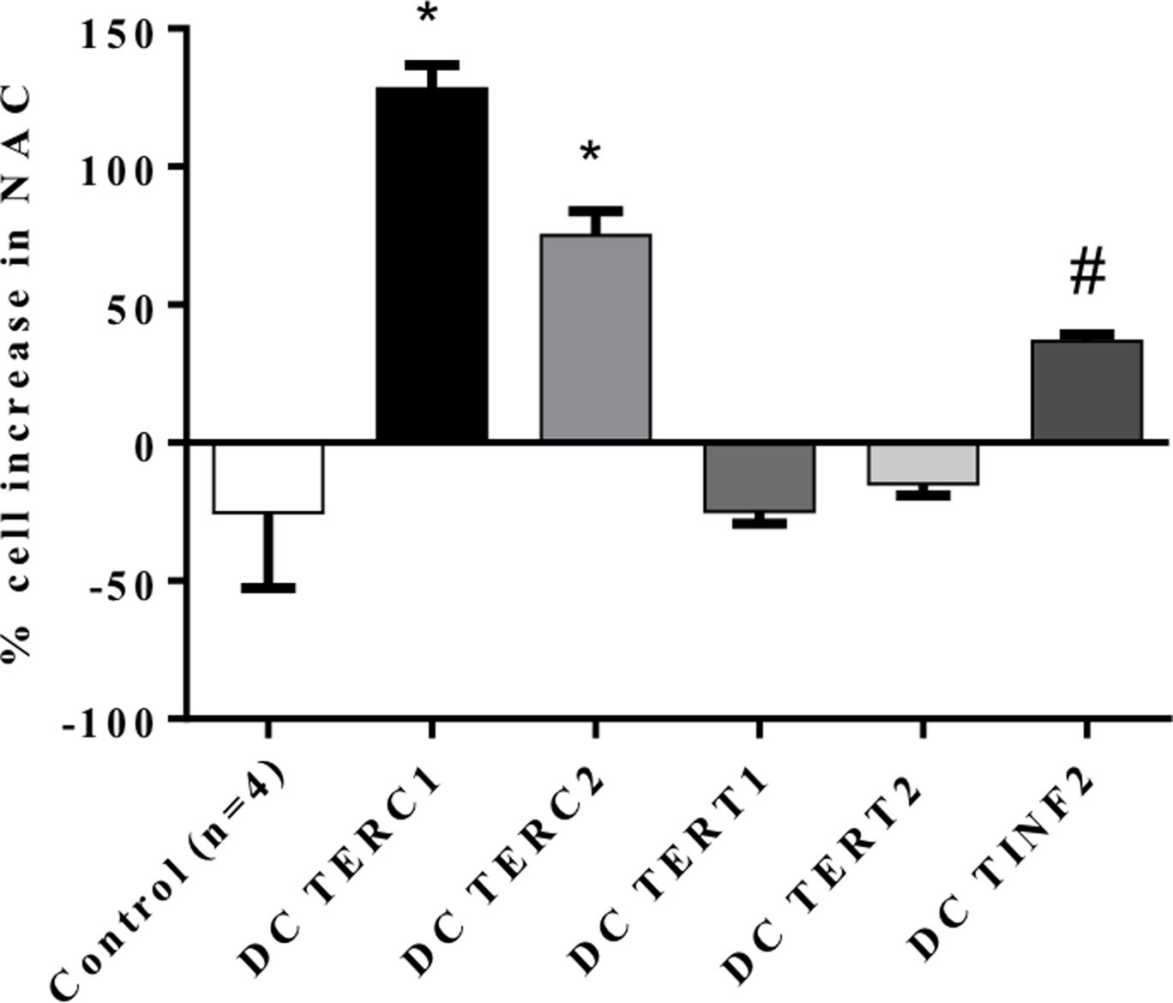
Effect of the antioxidant NAC on DC cells’ proliferation and ROS levels. Control and DC lymphocytes were cultured under routine conditions for five with daily supplementation of NAC (5mM). Cells were counted and graphed as the percentage change in cell number in NAC-supplemented cultures versus untreated controls after five days in culture. Error bars represent technical replicates within each sample. In comparison to control cells, statistical significance is reached in TERC and TINF2 samples (* indicates p<0.0001, # indicates p<0.002).

**Fig 6:**
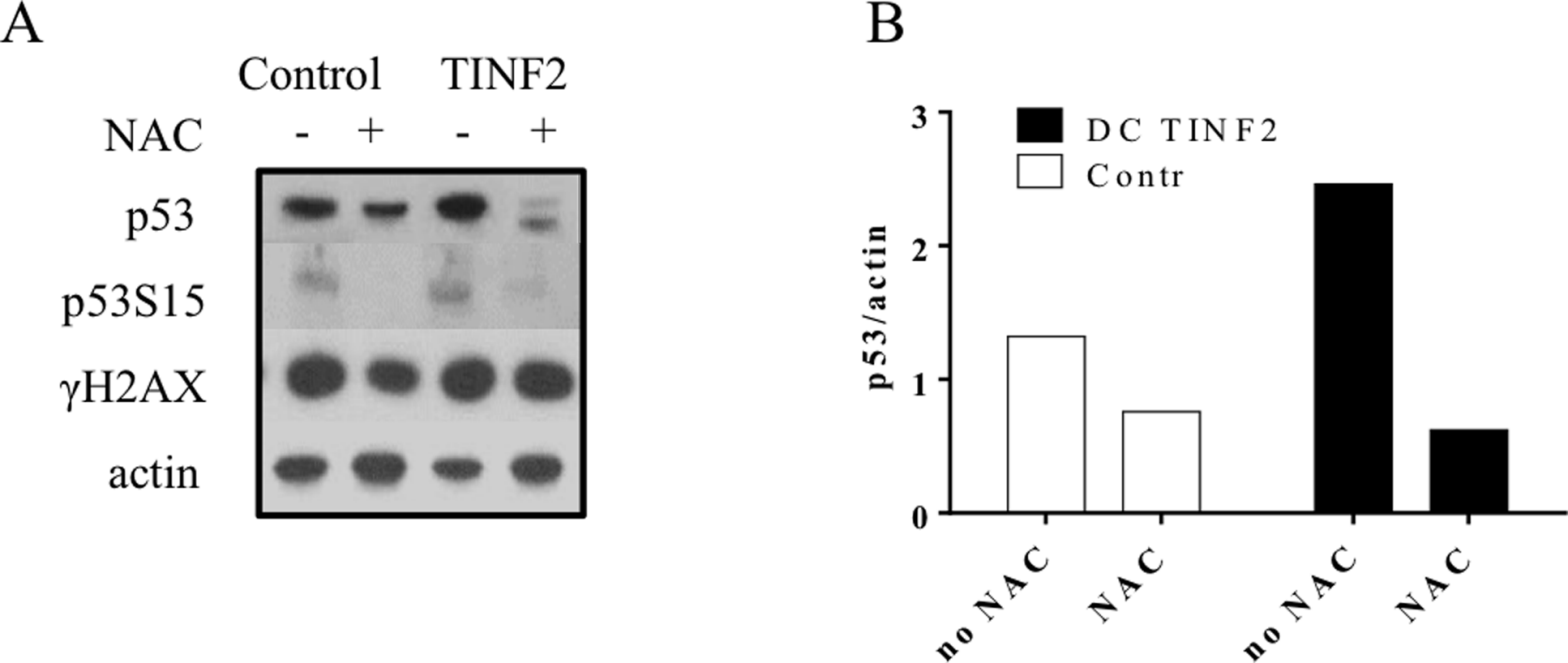
DDR markers post-NAC treatment in TINF2 cells. Control and TINF2 DC lymphocytes were cultured under routine conditions with 5mM NAC added daily. After five days, cells were collected and evaluated for p53 expression. (A) DC and control cells were evaluated by western blot for p53, p53 Ser15 and γH2AX by western blot in response to NAC. (B) Western blotting densitometry was calculated for p53.

**Fig 7:**
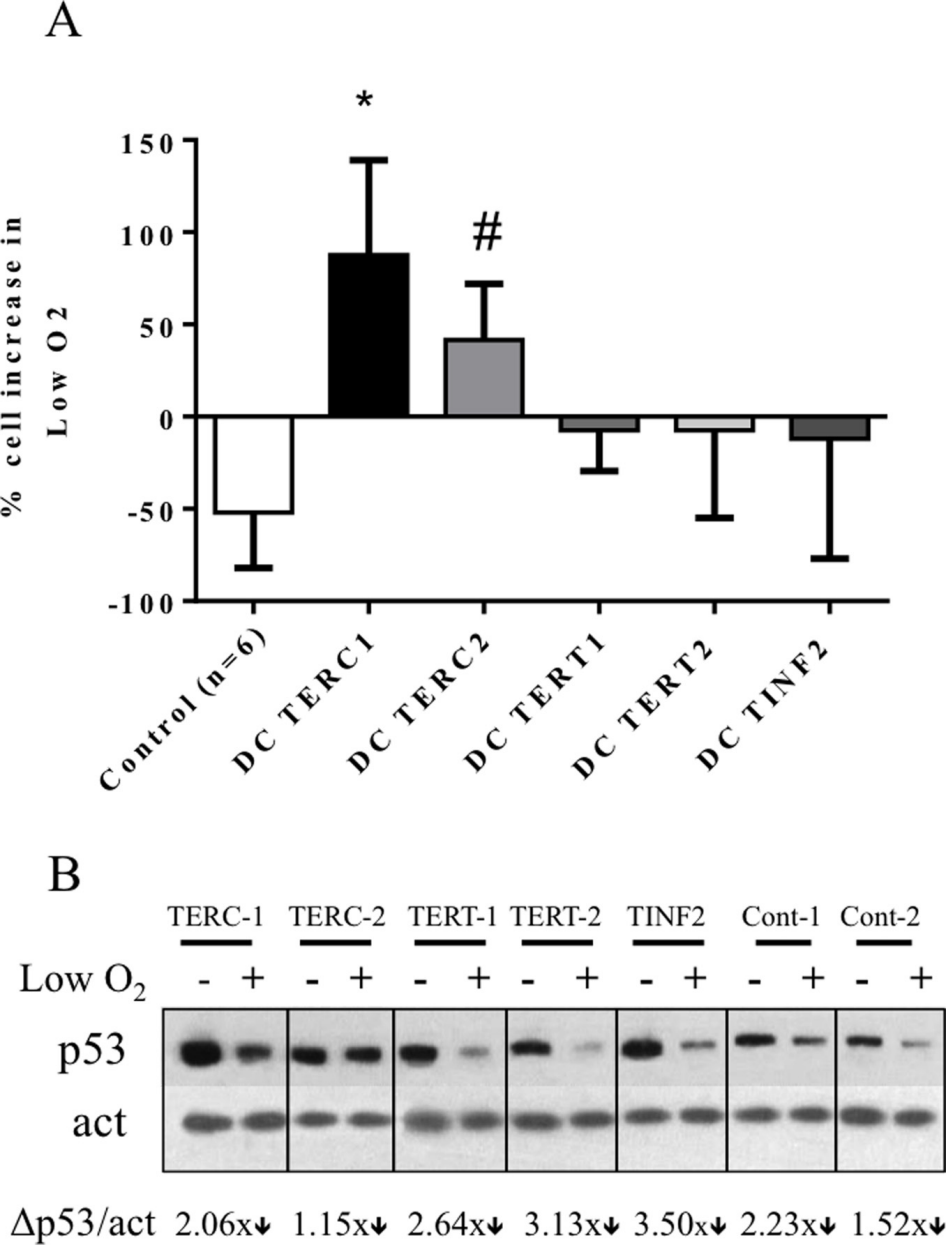
Effect of low oxygen on proliferation and p53 levels. Control and DC lymphocytes were cultured over four days and subsequently passaged to continue growth at normoxia (20% oxygen) or passaged into low oxygen condition (1%) for seven days. (A) Cell counts were performed using Nexcelom cell counter to evaluate proliferation. Data is presented as the percentage of total cells low versus ambient oxygen culture wherein ‘0’ is no change in proliferation. One of the TERC samples reached statistical significance (* indicates p = 0.026) while the second TERC sample increased in cell number but not to a statistically significant degree (# indicates p = 0.088). Error bars represent the standard deviation of two experimental replicates. (B) p53 protein levels were evaluated in cells cultured in ambient and low oxygen. Densitometry data calculating the fold-change between p53 levels at normoxia vs low oxygen is provide beneath the western blot (actin densitometry serves as internal control).

We have previously documented that treatment with the antioxidant NAC improved the proliferative capacity of *TERC* lymphocytes[14]. In this study, we carried out a similar set of experiments to ascertain whether NAC could prove beneficial to cells with other DC mutations and investigate potential underlying mechanisms. We treated DC cells for 5 days with NAC and found varying degrees of rescue in regards to NAC’s effect on proliferation. *TERC* cells benefitted the most (50–125% gain) whereas *TINF2* cells also increased in cell number over this period (~40%). Interestingly, *TERT* cell proliferation was slightly inhibited upon NAC supplementation, a finding similar to controls that saw either no change or slight inhibition (Fig 5). Compared to controls, these findings are statistically significant (TERC patient 1 and 2: p<0.0001; TINF2 patient: p<0.002). This data suggests that NAC may be beneficial to stimulate proliferation in DC cells. We next investigated whether the improved proliferation was related to a modified DDR. *TINF2* cells were cultured in the presence/absence of NAC and tested for the expression of DDR-related proteins (Fig 6). Total p53 and serine-15 phosphorylated p53 were both significantly decreased with NAC supplementation (Fig 6A) with total p53 decreasing approximately 5-fold (Fig 6B). This suggests that improved proliferation may be related to suppression of DDR. To further test the effects of modulating the oxidative environment, cells were grown in low oxygen (1%). Decreased oxygen tension has been characterized to improve proliferation in a number of primary cells[25, 26], and a previous report by our group demonstrated such an effect in *TERC* fibroblasts[17]. *TERC* lymphocytes, like the fibroblasts, revealed a growth advantage in low oxygen (50–125% improvement; Fig 7A) yet had a negligible effect on *TERT/TINF2* cells (statistical significance reached for one *TERC* patient, p = 0.026; second TERC patient p = 0.088). Next, cell lysates were collected from cultured cells in the above conditions and assayed for p53 expression via western blot and quantified by densitometry (Fig 7B). Interestingly, reduced oxygen tension decreased the expression of p53 in DC cells however the difference was most pronounced in the cells without improved proliferation (*TERT* and *TINF2*, controls). Together, these results suggest that decreasing oxygen tension decreases p53 protein levels in all lymphocytes however whether proliferative gains made in low oxygen is due to p53 diminution is not entirely clear. Thorough examination of p53 post-translational modifications in DC cells grown while manipulating oxygen tension will be of interest. In summary, NAC and low oxygen proved beneficial for proliferation in a subset of DC cells that may be related to attenuating a DDR. Nonetheless, given the importance of p53 in DDR and the striking decrease of ROS found when expressing p53 shRNA in DC fibroblasts[17], decreasing p53 activity may be one way DC cells are afforded a growth benefit via NAC and perhaps low oxygen approaches.

## Discussion

DC is a telomere dysfunction disorder with a variety of clinical manifestation in highly proliferative tissues, including skin, gastrointestinal tract, and the hematopoietic system. To date, mutations in twelve genes have been linked to a DC phenotype, yet 40% of DC cases lack an identifiable mutation. Genetic diversity and clinical complexity have made genotype-phenotype correlation in DC challenging[2, 27, 28]. Regarding the most prevalent DC mutations, in general, it appears that *DKC1* and *TINF2* patients acquire the earliest and most severe DC pathologies, followed by *TERC* and finally *TERT*[2]. Variable presentation of the DC phenotpe is likely due to the underlying mechanisms of the telomere/telomerase defect. For instance, males with mutations in *DKC1* have dramatically decreased telomerase deficiency due to the fact that X-linked mutations lead to failed telomerase biogenesis via TERC trafficking and accumulation[29]. DKC1 pathogenesis may also be related to functions not directly related to telomerase or telomere maintenance (rRNA pseudouridylation[30] and IRES-specific defects[31]). *TERC* mutations, on the other hand, are typically haploinsufficient whereupon telomerase activity is diminished by 50% due to mutations that knockout a single, autosomal allele[32]. *TERT* mutations reveal variable penetrance and may carry some latent activity that appears to decrease the level of severity[2, 33]. *TINF2* mutations appear to be fundamentally different[34]. TINF2 is not a member of the telomerase complex but acts as a scaffolding protein within shelterin that interacts with TRF1, TRF2, POT1[35, 36]. Mutations within *TINF2* likely disrupt the shelterin complex as well as prevent the recruitment telomerase[37]. Together, the disruption of these two key roles may create uniquely potent DC mutations resulting in increased DDR and decreased access to the telomere by telomerase to extend shortened/dysfunctional telomeres. This may explain the heightened DDR and ROS found here in *TINF2* cells and phenotypic severity found in *TINF2* patients. As an extension of these findings, experiments carried out by our lab compared the clinical and molecular features of different DC genotypes (summarized in Table 1). We hypothesize that short telomeres signal a “stress phenotype”, as supported by elevated DDR and ROS, and this is a central mediator between shortened telomeres and of the observed pathology in DC.

The generalized severity of DC patients’ phenotypes appears to be exemplified by the underlying DDR and ROS within their cells. In our study, an increase in steady-state levels was noted for all DC lymphocytes (Fig 2A–2D), in accordance with our previous observations for DC *TERC* cells[14]. Of note, multiple p53 bands appeared in Figs 2C and 6A western blots. The presence of multiple p53 bands has been reported by others and is likely the result of post-translational modification alterations and/or changes in splice variants expressed[38–40]. Further investigation will be required to better understand the molecular changes that p53 is undergoing as it relates to DC. The magnitude of the p53 increase varied among DC mutations with the highest found in *TERC* and *TINF2* cells while *TINF2* cells appear to have a very robust DDR compared to *TERT* cells (Fig 2C). It is worth noting that our patients carrying the *TERC* and *TINF2* mutations presented with the most advanced disease (Table 1), supporting the hypothesis that DDR may represent a biomarker for disease severity. The relationships between telomere dysfunction, DDR, and p53 activation is well established[7, 41–43]. Shortened telomeres engage DDR by activating p53, which is a key determinant in cell fate decisions. This activation likely includes the mobilization of the cyclin-dependent kinase inhibitor p21, which we previously found increased in DC *TERC* lymphocytes[14]. Attenuating p53 through different mechanisms rescues some of the defects associated with short telomeres, further supporting the role of p53 in telomere-related pathologies[17, 44]. Despite notable differences in steady-state p53 levels, post-irradiation p53 levels were still increased in DC cells however the magnitude of the increase was greater in controls (Fig 2A and 2B). Comparable observations were noted by others whereupon steady-state DNA damage markers (γH2AX, 53BP1, ATM, Chk2, etc.) were elevated in DC human lymphocytes and fibroblasts compared to normal cells[13, 22]. These groups found an increased DDR in DC cells in response to the cytotoxic agents bleomycin and etoposide, yet the magnitude was not as pronounced as in the controls. The consistent increase in p53 of all three mutations suggests a convergence of short telomere signaling on this tumor suppressor regardless of the underlying mutation. Although further work will be required, it is tempting to speculate that *TINF2* mutations may mount a more robust DDR, distinct from other DC mutations, that could alter the underlying DC pathology as evidenced by the presence of retinopathy in this particular patient.

A number of disorders associated with telomere shortening (including cancer, bone marrow failure syndromes and aging) have a concomitant increase in ROS or disturbances in oxidative pathways, suggesting that the related underlying pathologies may be due in part to redox modulation[45, 46]. Equilibration of ROS within the cell is fine-tuned by anti- and pro-oxidant systems, whereupon reduced or excessive ROS each can be detrimental. In addition, ROS can promote telomere attrition suggesting a potential feedback loop to sustain elevated ROS and favor entry into senescence[43]. Unlike the DDR, all DC cells maintained a striking increase in ROS upon irradiation and exposure to peroxide compared to controls (Figs 3 and 4) suggesting that such cellular insults may be more dramatic in short telomere cells and may be less tolerated in *TERC* and *TINF2* patients. This also suggests that ROS-related responses in DC cells are deregulated in a way not found in controls. While the molecular mechanisms underlying the growth disadvantage in DC cells is not completely understood, it is tempting to speculate that the contribution of aberrant DDR activation and/or sustained elevated oxidative is significant. However this is complicated by the fact that ROS can be detrimental to cells[47, 48] yet required for optimal T-cell activation through the production of IL-2 production via increased peroxide [49, 50]. Use of the antioxidant NAC or low oxygen provided a means to test the causative effect of elevated ROS in DC cells. We found that NAC improved proliferation in DC cells harboring the highest levels of ROS (*TERC*, *TINF2*) yet had a negligible or negative effect on proliferation in cells with lower ROS (*TERT* and control cells) (Fig 5). Low oxygen, on the other hand, proved beneficial for only *TERC* cells and had no effect on *TERT* and *TINF2* cells. Interestingly, low oxygen inhibited control cells suggesting that the effect of low oxygen differs between control and DC cells. Further experiments will need to be carried out to determine if specific subsets or cellular pools of ROS may be diminished upon NAC/low oxygen treatment. In addition, adjusting the concentration of NAC commensurate with ROS levels may prove beneficial for each DC mutation as ROS levels varied among the three mutations tested here. Interestingly, total p53 levels could be diminished in DC cells by NAC and low oxygen (Figs 6 and 7) leading to modest proliferative gains in some DC cells, suggesting that proliferative benefits gained by an antioxidant approach may be related to suppressing DDR. Consistently elevated ROS observed in DC lymphocytes may imply either 1. pro-oxidant pathways are deregulated or 2. antioxidant countermeasures are underperforming.

## Summary

In summary, the current study was undertaken to investigate DDR and ROS levels within DC cells of varying mutations. We demonstrated that p53 was upregulated in stimulated lymphocytes of all DC mutations tested, with robust mobilization of DDR in TINF2 cells. In addition, all DC lymphocytes demonstrated elevated levels of ROS in routine culture conditions and after irradiation. Thus, a “stressed phenotype” comprised of increased DDR/ROS is most likely a common attribute of cells with short telomeres. Attempts to decrease ROS (via NAC or low oxygen conditions) ameliorated cell proliferation in subsets of DC cells and decreased p53 protein levels. Data presented here suggests that disease phenotype may correlate with ROS and DDR responses, with the highest levels found in *TINF2*, followed by *TERC* and finally *TERT*. underlying DDR/ROS levels presenting a potential biomarker. Finally, clinical trials will be needed to determine whether pharmacological interventions aimed at reducing ROS will have a clinical benefit for the systemic manifestations of DC.
